# Crystal structure analysis, Hirshfeld surface analysis and DFT calculations of a one-dimensional neutral manganese(II) coordination polymer [(CH_3_)_3_NH][MnCl_2_(H_2_O)_2_]Cl

**DOI:** 10.1107/S2056989026010765

**Published:** 2026-10-30

**Authors:** Zhangran Gao, Jiawei Li, Jinwu Zheng, KaiXue Jie

**Affiliations:** a School of Electrical and Information Engineering Fujian University of Technology Fuzhou 350118 People’s Republic of China; b School of Science Qingdao University of Technology,Qingdao 266250 People’s Republic of China; Universidad de la República, Uruguay

**Keywords:** crystal structure, chloride-bridged chain, hydrogen bonding, Hirshfeld surface analysis, DFT calculations

## Abstract

The crystal structure of tri­methyl­ammonium manganese(II) chloride dihydrate or *catena*-poly[tri­methyl­ammonium [[di­aqua­manganese(II)]-di-μ-chlorido] chloride], {[(CH_3_)_3_NH][MnCl_2_(H_2_O)_2_]Cl}_*n*_ (**1**), originally reported by Caputo *et al.* (1976 ▸). *Acta Cryst.* B**32**, 2639–2642], has been redetermined at 100 K. The structure retains the ortho­rhom­bic space group *Pnma* and the coordination connectivity of the previously reported room-temperature structure. Each Mn^II^ ion lies on an inversion centre and is coordinated by four bridging chloride ligands and two mutually *trans* water mol­ecules. The resulting edge-sharing MnCl_4_(H_2_O)_2_ octa­hedra form neutral chains parallel to the *b* axis, with tri­methyl­ammonium cations and non-coordinating chloride anions situated between the chains. The measured unit-cell parameters, bond lengths and bond angles are in good agreement with the room-temperature determination reported by Caputo *et al*. In the crystal, O—H⋯Cl and N—H⋯Cl contacts link the inorganic and organic components. Hirshfeld surface analysis identifies H⋯Cl/Cl⋯H contacts as the largest surface-area contribution. Crystal morphology predictions and periodic density functional theory (DFT) calculations provide supplementary descriptions of the crystal habit and electronic response.

## Abstract 2

The low-temperature redetermination of tri­methyl­ammonium manganese(II) chloride dihydrate confirms neutral one-dimensional chains of edge-sharing *trans*-[MnCl_4_(H_2_O)_2_]_*n*_ octa­hedra extending along the *b*-axis direction. O—H⋯Cl and N—H⋯Cl hydrogen bonds are present in the crystal.

## 1. Chemical context

Chloride-bridged manganese(II) compounds provide structurally simple examples of extended coordination assemblies. In these materials, bridging chloride ligands can connect Mn^II^ centres into chains, while coordinated water mol­ecules and organic ammonium ions participate in hydrogen-bonding contacts between the inorganic and organic components. Their structural description therefore requires consideration of both the metal–ligand connectivity and the inter­actions between neighbouring chains.
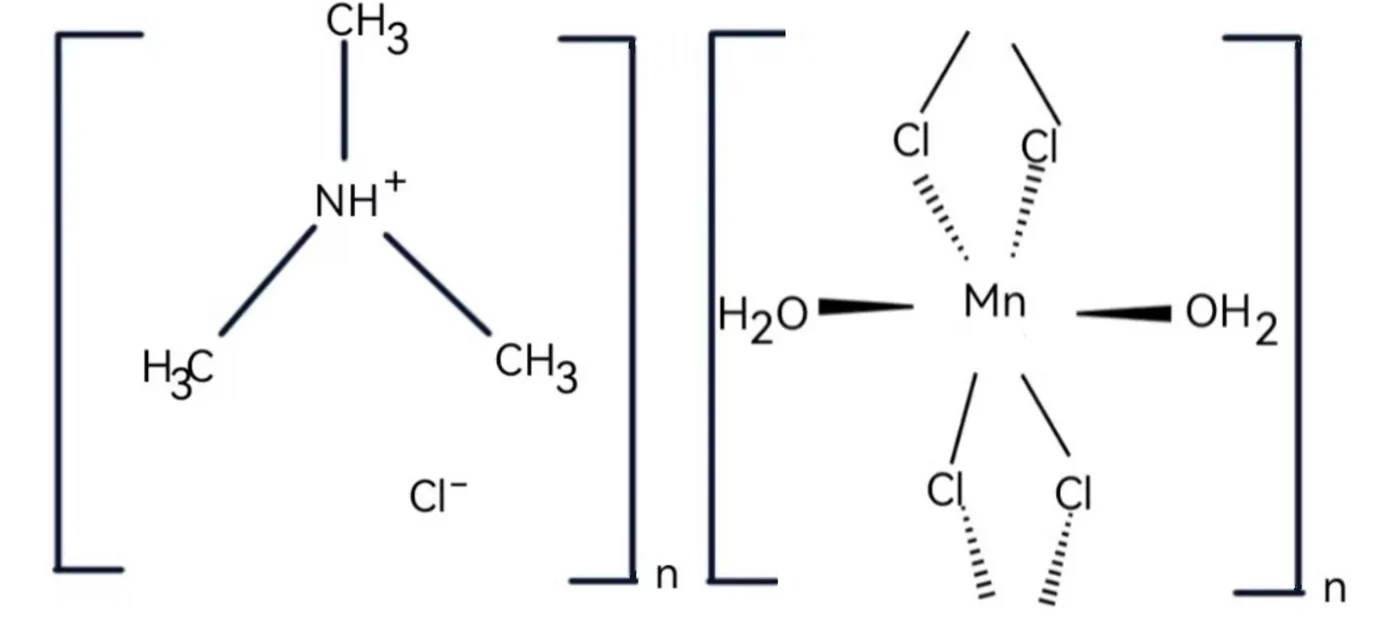


The crystal structure of tri­methyl­ammonium manganese(II) chloride dihydrate, [(CH_3_)_3_NH]·[MnCl_2_(H_2_O)_2_]·Cl, was established by Caputo *et al.* (1976 ▸). Their study identified neutral chains of edge-sharing MnCl_4_(H_2_O)_2_ octa­hedra, together with tri­methyl­ammonium cations and discrete chloride anions between the chains. It also described hydrogen bonding between the chains and discussed the structure in relation to the magnetic behaviour of this family of compounds. The identity and principal structural features of the title compound are therefore already established.

The present work reports an independent determination of this structure at 100 K. Hirshfeld surface analysis is used to describe the relative surface-area contributions of different contact types. A geometrical crystal morphology model and periodic density functional theory calculations are included as complementary analyses.

## 2. Structural commentary

**Figure 1:**
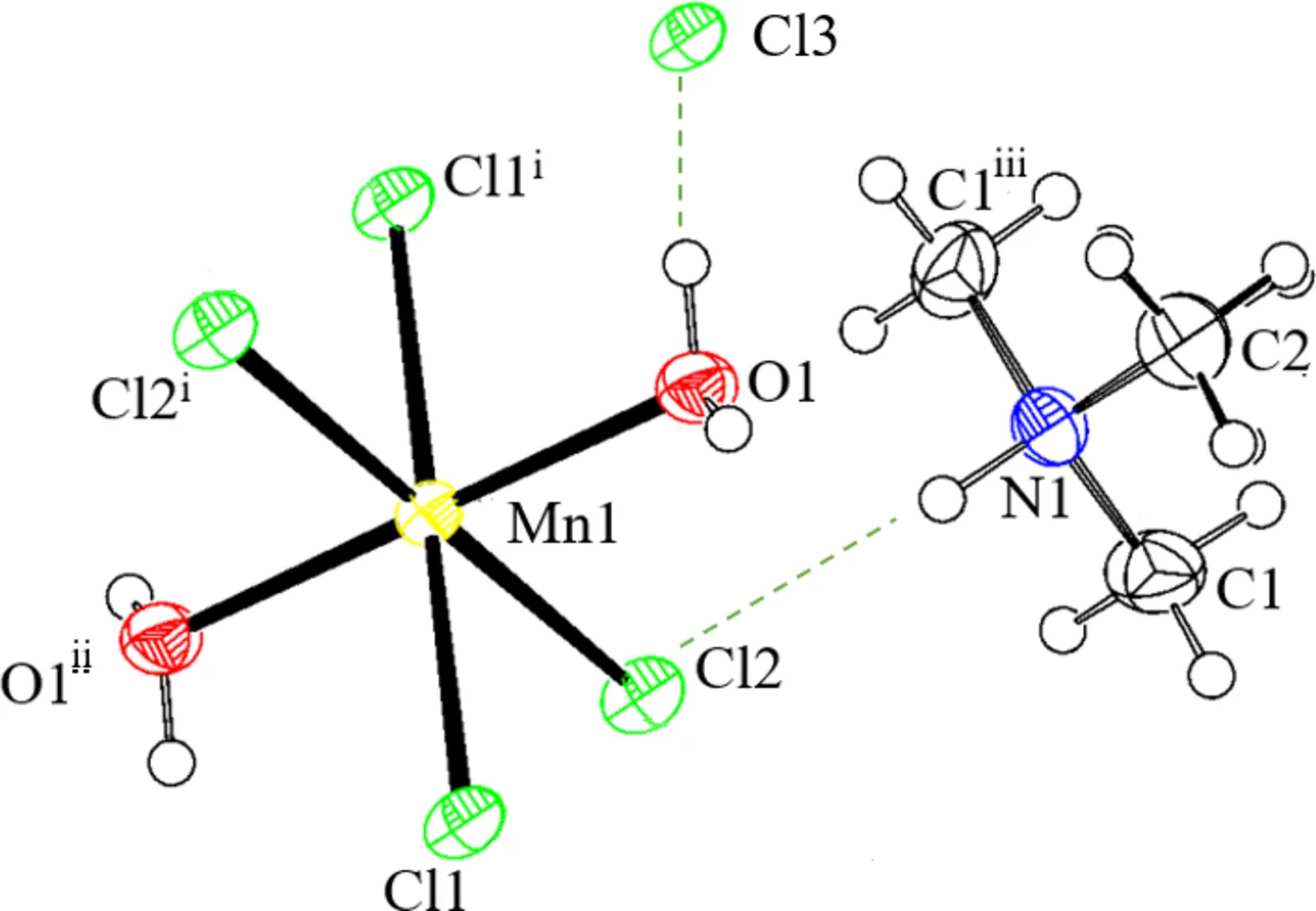
View of part of the [MnCl_2_(H_2_O)_2_]_n_ chain with one tri­methyl­ammoium and one chloride ions, showing 50% probability displacement ellipsoids. Symmetry codes: (i) 1 − *x*, −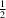 + *y*, −*z* + 1, (ii) 1 − *x*, 1 − *y*, 1 − *z*, (iii) *x*, 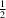 − *y*, *z*.

The crystal structure of the title compound was previously reported by Caputo *et al.* (1976 ▸) at room temperature. The title compound crystallizes in the ortho­rhom­bic space group *Pnma*. The Mn^II^ ion occupies an inversion centre and has a distorted octa­hedral coordination environment comprising four bridging chloride ligands and two mutually trans water mol­ecules (Fig. 1 ▸). The two independent Mn—Cl distances are 2.5277 (4) and 2.5845 (4) Å, and the Mn—O distance is 2.1929 (11) Å. Adjacent octa­hedra share chloride-bridged edges, producing neutral one-dimensional chains parallel to the b axis. The two independent Mn—Cl—Mn bridging angles are 94.982 (19) and 92.269 (19)°. Tri­methyl­ammonium cations and non-coordinating chloride anions occupy the regions between the chains. The space group and coordination connectivity are unchanged. The present unit-cell volume of 1030.81 (12) Å^3^, together with the bond-length data, is consistent with the published room-temperature values.

**Figure 2:**
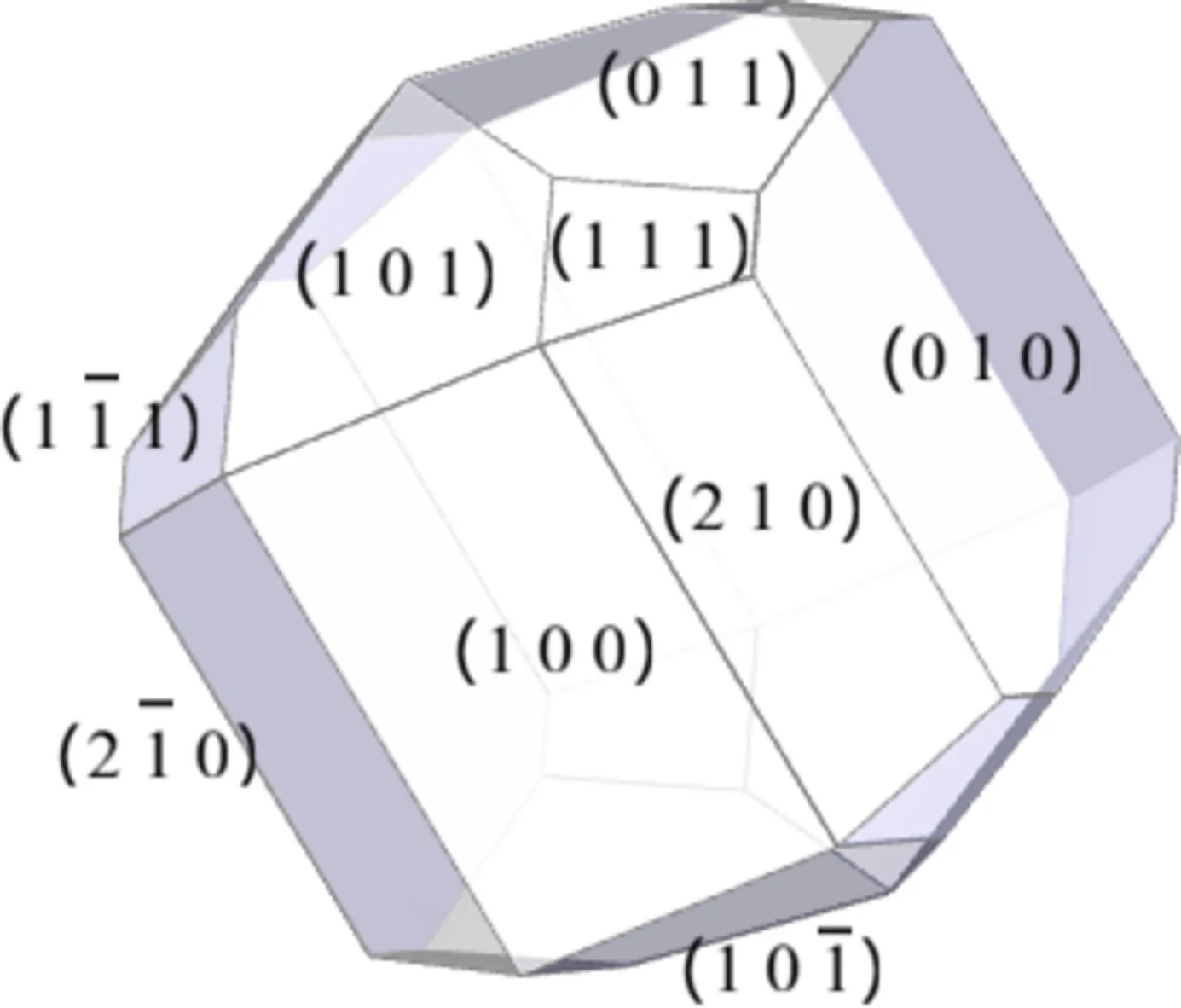
Calculated crystal-face model of the title compound, showing the Miller indices of the labelled faces.

The crystal morphology of the title compound was predicted using the Bravais–Friedel–Donnay–Harker (BFDH) model implemented in *WinXMorph* (Kaminsky, 2005 ▸). The predicted morphology exhibits a faceted polyhedral shape, with indexed faces including (100), (010), (210), (011), (101) and (111) (Fig. 2 ▸). The BFDH model provides a geometrical prediction of crystal morphology based on lattice parameters and crystal symmetry (Donnay & Harker, 1937 ▸). Accordingly, the resulting model illustrates the possible crystal habit without explicitly accounting for surface energetics or the effects of crystallization conditions.

## 3. Supra­molecular features

In the crystal packing, parallel neutral {[Mn(μ_2_-Cl)_2_(H_2_O)_2_]}_*n*_ chains extend along the *b*-axis direction, with [(CH_3_)_3_NH]^+^ tri­methyl­ammonium cations and free chloride anions (Cl3) occupying the voids between adjacent chains. Both species form hydrogen bonds with the inorganic chains, including O—H⋯Cl and N—H⋯Cl inter­actions (Table 1 ▸). The coordinated water mol­ecules act as hydrogen-bond donors, forming O—H⋯Cl hydrogen bonds with the non-coordinating chloride anions (Cl3). The [(CH_3_)_3_NH]^+^ tri­methyl­ammonium cations also serve as hydrogen-bond donors, forming N—H⋯Cl hydrogen bonds with the bridging chloride ligands of the inorganic chains. Hydrogen bonds involving coordinated water mol­ecules and non-coordinating chloride anions were also reported in the room-temperature structure (Caputo *et al.*, 1976 ▸).

## 4. Hirshfeld surface analysis

**Figure 3:**
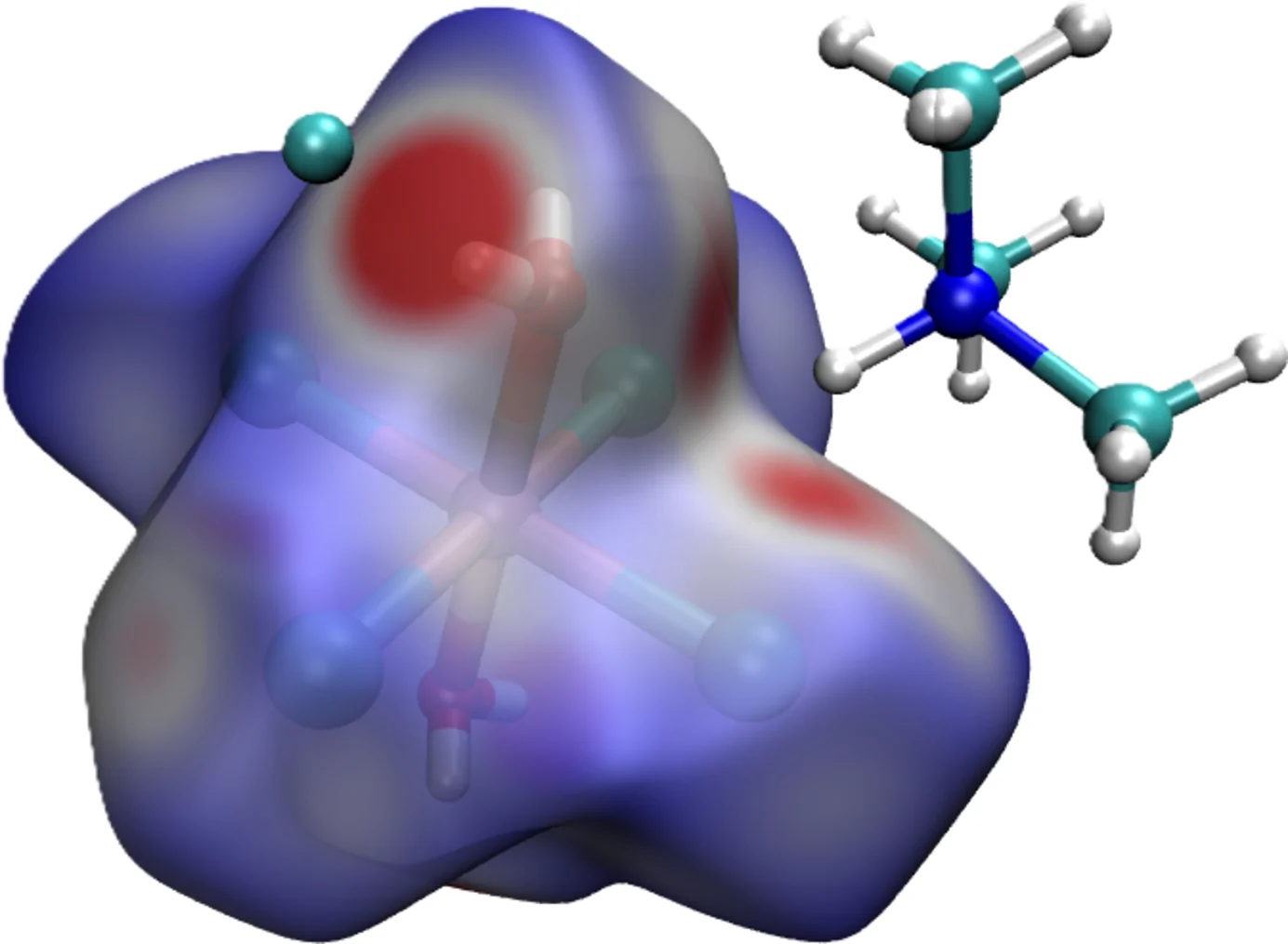
Hirshfeld surface of the title compound, mapped over *d*_norm_.

**Figure 4:**
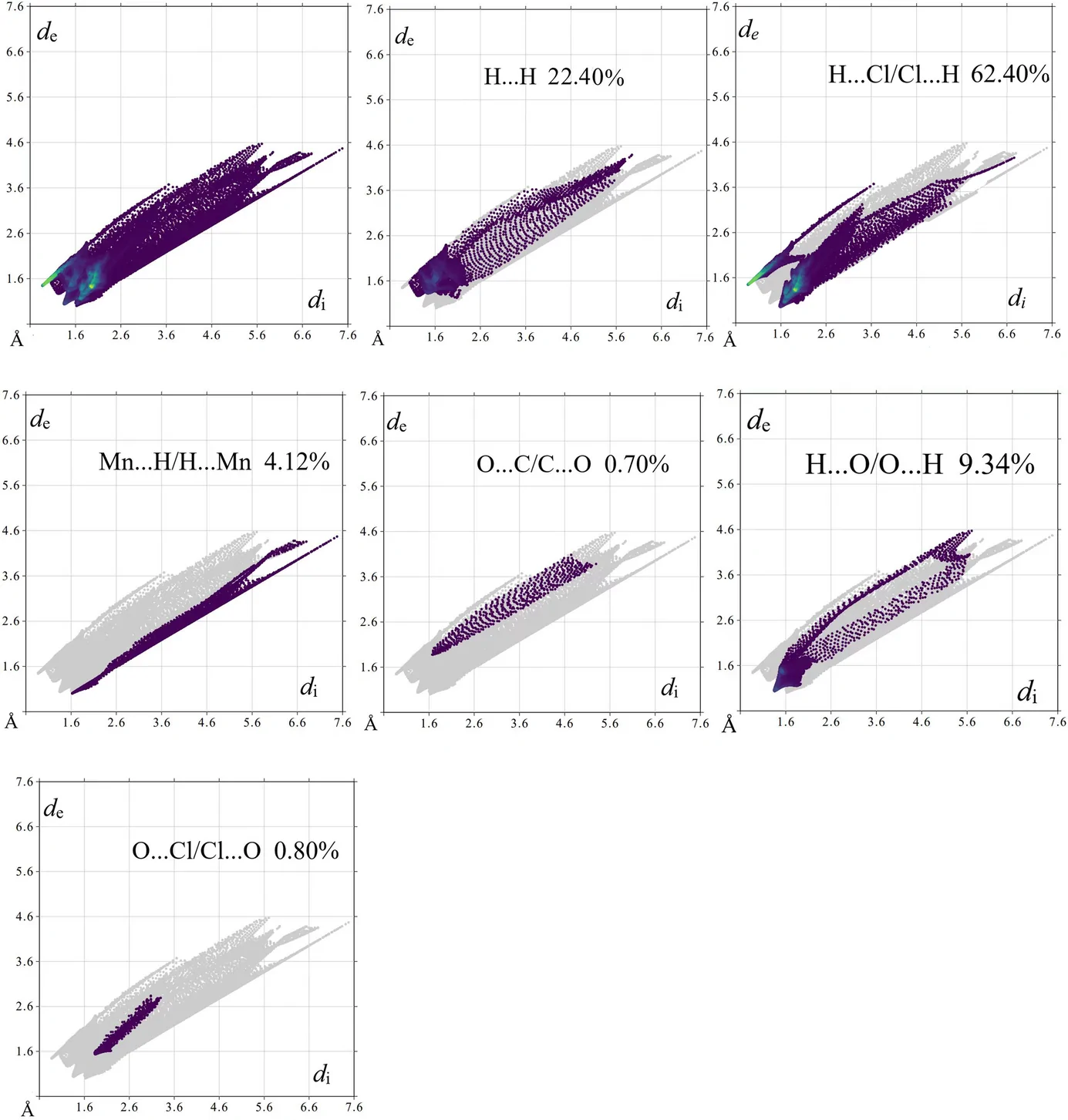
Two-dimensional fingerprint plots for the title compound.

To further investigate the inter­molecular contacts involving the tri­methyl­ammonium cations, free chloride anions, and the one-dimensional inorganic chain framework, a Hirshfeld surface analysis was undertaken (Fig. 3 ▸) and the corresponding two-dimensional fingerprint plots (Fig. 4 ▸) were generated for the title compound. The Hirshfeld surface was calculated from the crystallographic information file using *Multiwfn* (Lu & Chen, 2012 ▸) and visualized with *VMD* (Humphrey *et al.*, 1996 ▸). This analysis provides a graphical and qu­anti­tative description of the close contacts associated with the selected structural unit in its crystalline environment.

The Hirshfeld surface mapped over the normalized contact distance provides an intuitive visualization of the distribution of inter­molecular contacts. Red regions indicate contacts shorter than the sum of the corresponding van der Waals radii, white regions represent contacts with distances close to this sum, and blue regions correspond to longer contacts (Spackman & Jayatilaka, 2009 ▸). Prominent red spots are observed in the surface regions associated with the O—H groups of the coordinated water mol­ecules and the N—H group of the tri­methyl­ammonium cation. Based on the crystallographic environment, these features can be assigned to the O—H⋯Cl3 contacts involving the free chloride anions and the N—H⋯Cl contacts involving the bridging chloride ligands of the one-dimensional chains, respectively. These surface features are consistent with the hydrogen-bonding contacts identified from the single-crystal structure.

The H⋯Cl/Cl⋯H contacts make the largest contribution to the Hirshfeld surface, accounting for 62.40% of the total surface area, indicating the frequent occurrence of short hydrogen–chloride contacts in the crystal. This observation is consistent with the chloride-rich crystalline environment of the compound and with the crystallographically identified O—H⋯Cl and N—H⋯Cl inter­actions. H⋯H contacts make the second-largest contribution, accounting for 22.40% of the Hirshfeld surface. Their broad and diffuse distribution in the fingerprint plot is characteristic of relatively non-directional close contacts, including those involving the methyl hydrogen atoms of the tri­methyl­ammonium cations. H⋯O/O⋯H contacts contribute 9.34%, whereas H⋯Mn/Mn⋯H, O⋯Cl/Cl⋯O, and O⋯C/C⋯O contacts account for 4.12%, 0.80%, and 0.70%, respectively. These smaller contributions indicate the presence of additional close contacts in the crystalline environment (Hashim *et al.*, 2021 ▸). Other contact types make only minor contributions and are not discussed individually.

Overall, the Hirshfeld surface and fingerprint analyses identify H⋯Cl/Cl⋯H as the most prevalent type of surface contact in the title compound. In conjunction with the O—H⋯Cl and N—H⋯Cl geometries determined crystallographically, these results support the importance of chloride-associated hydrogen-bonding contacts in the organization and consolidation of the crystal packing. However, the Hirshfeld surface percentages represent contact-area contributions and should not be inter­preted directly as relative inter­action energies or as independent evidence for the dimensionality of the supra­molecular assembly.

## 5. Density functional theory calculations

To gain further insight into the electronic structure and vibrational properties of the title compound, periodic spin-polarized density functional theory calculations were performed using *CASTEP* (Clark *et al.*, 2005 ▸) within the collinear spin formalism. The Perdew–Burke–Ernzerhof (PBE) exchange–correlation functional was employed together with the Tkatchenko–Scheffler dispersion correction. A Hubbard correction of *U* = 2.5 eV was applied to the Mn 3*d* orbitals. Norm-conserving pseudopotentials and a plane-wave energy cutoff of 750 eV were used. Brillouin-zone sampling employed a 1 × 1 × 1 Monkhorst–Pack k-point grid (Monkhorst & Pack, 1976 ▸). The self-consistent-field (SCF) energy convergence threshold was set to 2.0 × 10^−6^ eV per atom, and a density mixing scheme was used for electronic minimization. The experimentally determined crystal structure was used as the starting model for geometry optimization, and the optimized structure was subsequently used for single-point electronic-structure calculations.

**Figure 5:**
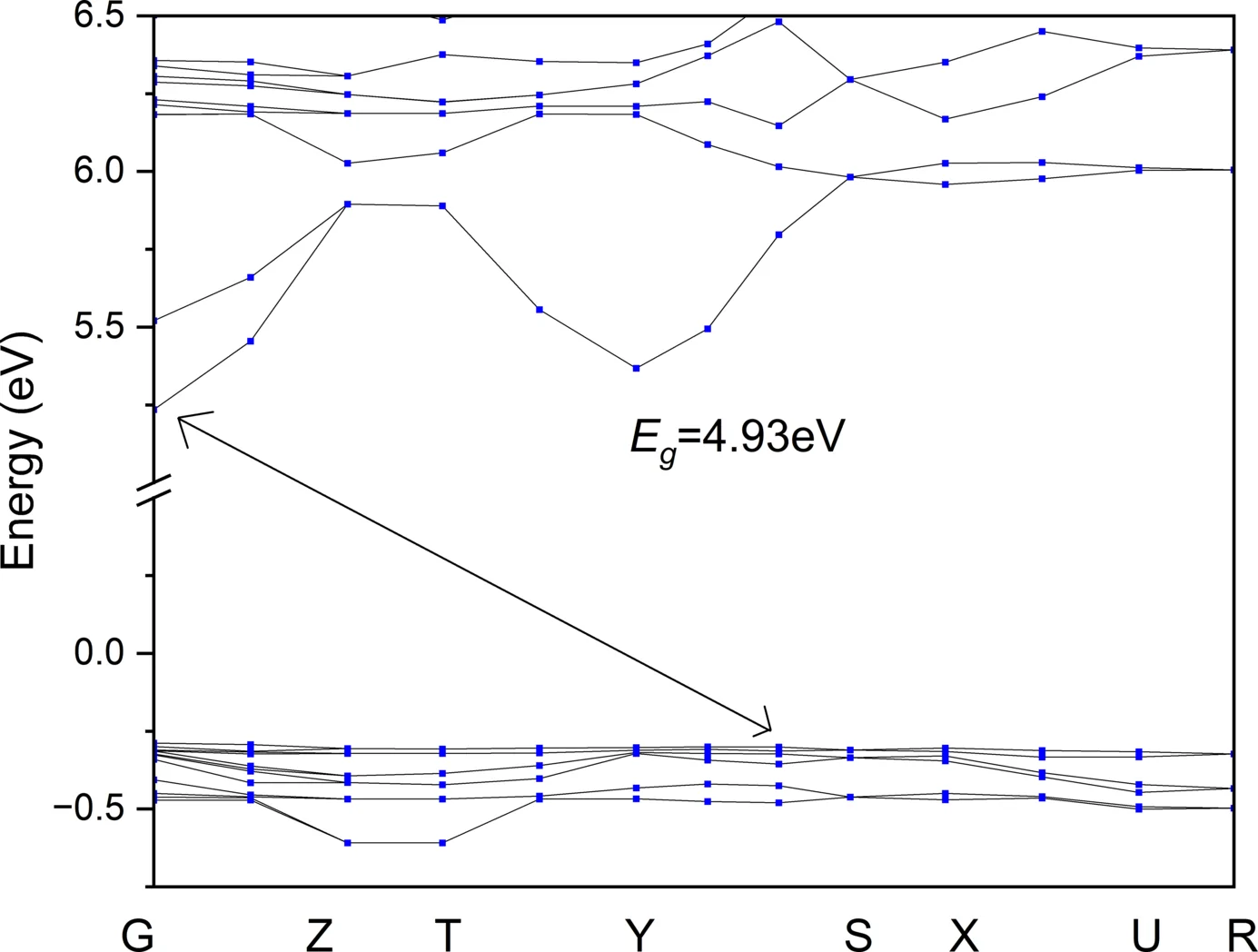
The band structure of the title compound.

The calculated electronic band structure along the high-symmetry path G–Z–T–Y–S–X–U–R is shown in Fig. 5 ▸. At the PBE+U level of theory, the compound exhibits a wide band gap (*E*_g_ = 4.93 eV). Both the valence and conduction bands are remarkably flat across the entire Brillouin zone, reflecting the weak inter­chain electronic coupling and highly localized character of the frontier electronic states, which is consistent with a mol­ecular ionic crystal dominated by electrostatic and hydrogen-bonding inter­actions. Notably, the flat band feature does not mean complete electronic isolation of Mn^II^ centres along the chain: superexchange inter­actions between adjacent Mn^II^ ions can still be mediated by the bridging chloride ligands, as widely demonstrated by magnetic studies on the isostructural [(CH_3_)_3_NH]·MnCl_3_·(H_2_O)_2_ family (Bhatia *et al.*, 1976 ▸) .

**Figure 6:**
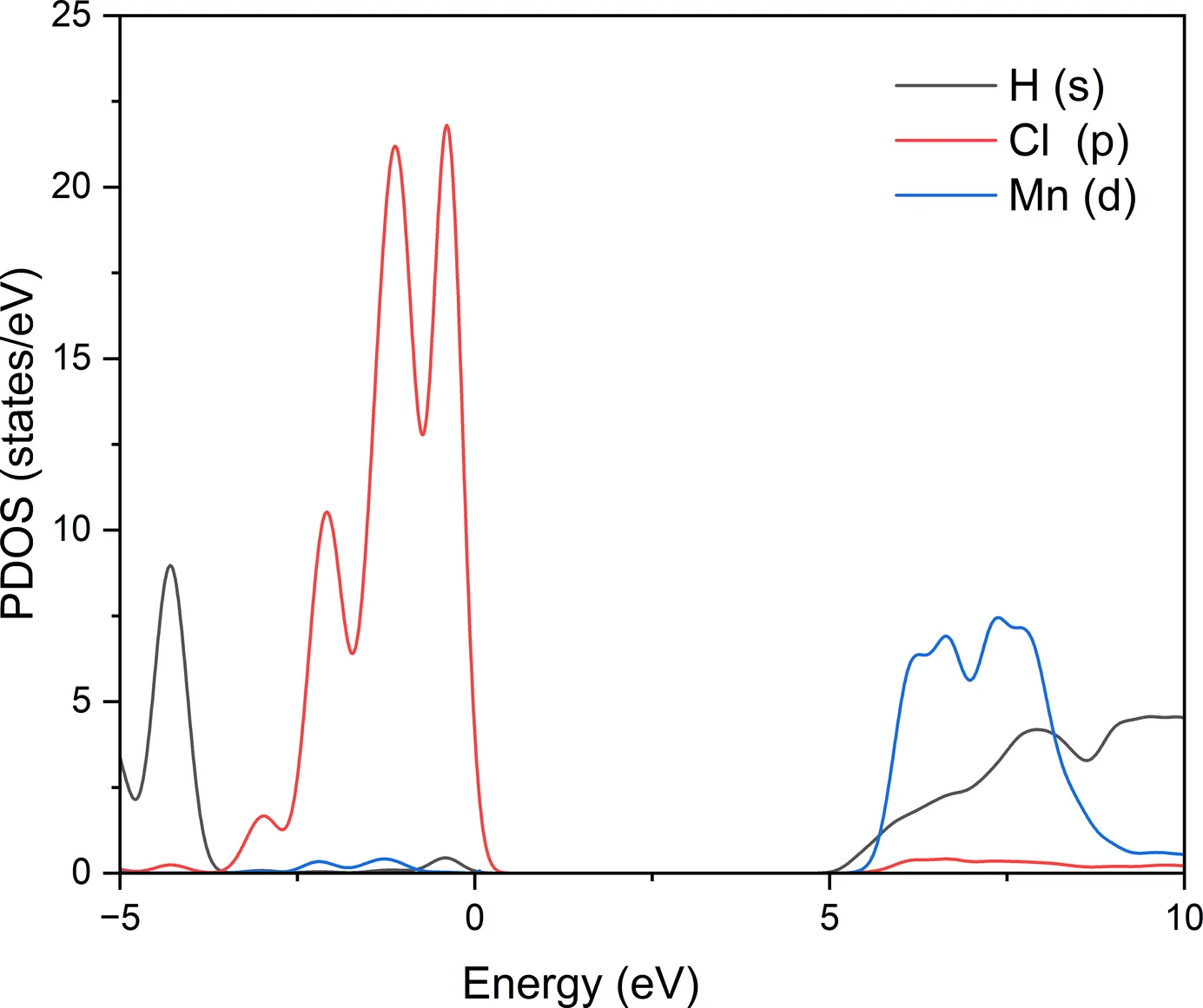
Partial density of states of the title compound.

The partial density of states (PDOS, Fig. 6 ▸) reveals that the upper valence band (−5 to 0 eV) is dominated by Cl 3*p* states, while the conduction band shows mixed *s*, *p* and minor Mn 3*d* contributions.

**Figure 7:**
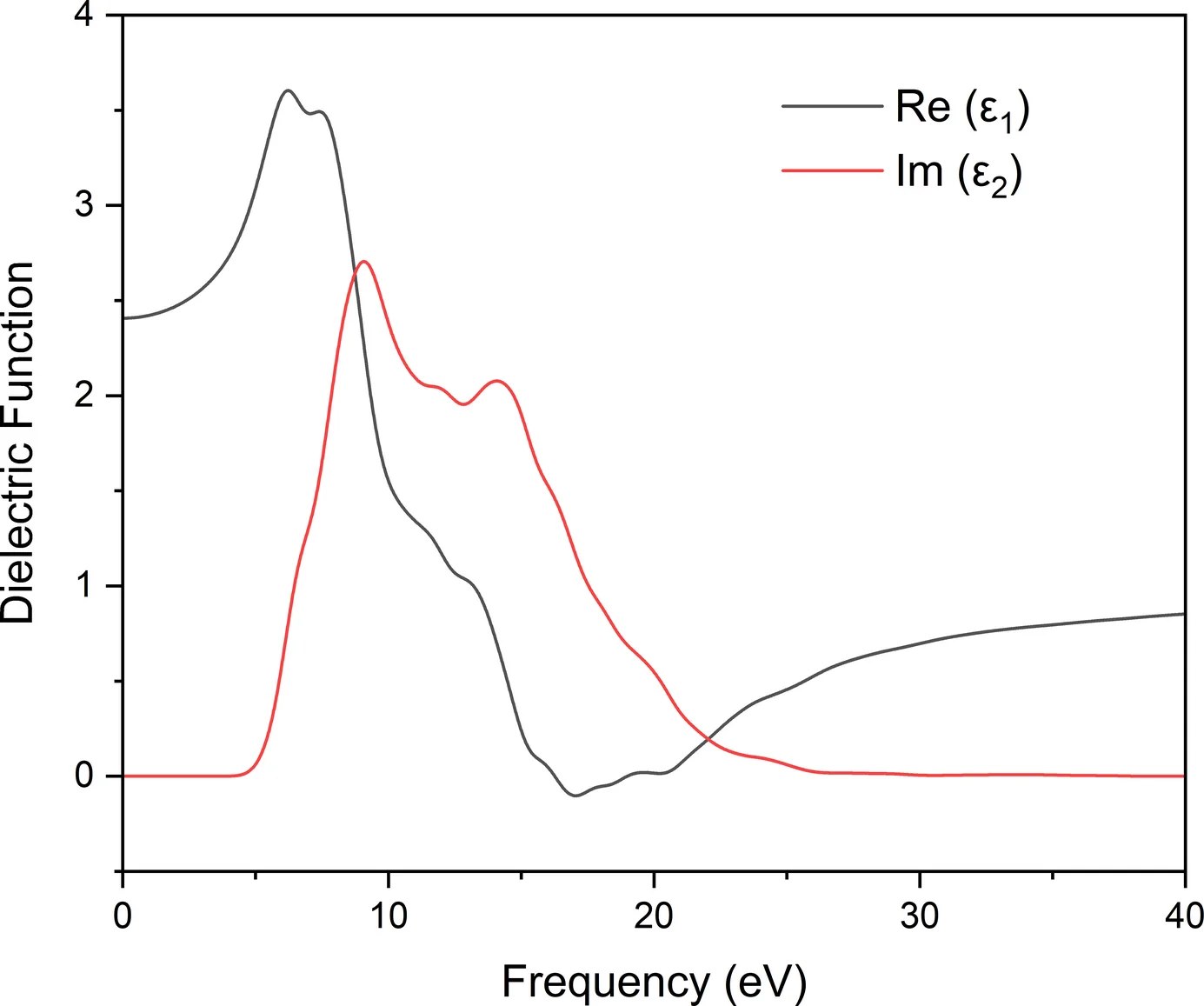
Calculated complex dielectric function of the title compound.

The calculated complex dielectric function of the title compound for [100] polarization is presented in Fig. 7 ▸. The imaginary part ɛ_2_(ω), which is directly related to the optical absorption probability, shows an absorption onset at approximately 5.0 eV, consistent with the wide-band-gap insulating character derived from the band structure calculation. The strongest absorption peak centred at *ca.* 8.7 eV is assigned to ligand-to-metal charge transfer (LMCT) transitions from the Cl 3*p*-dominated upper valence band to Mn 3*d* states in the conduction band, in full agreement with the partial density of states analysis.

The real part ɛ_1_(ω) displays typical normal dispersion in the transparent region below the absorption edge, with a static dielectric constant ɛ_1_(0) at the zero-frequency limit. This moderate value reflects the mol­ecular ionic nature of the crystal, where electronic polarization is largely localized within the one-dimensional Mn–Cl chains and discrete organic cations, with only minor contributions from weak inter­chain hydrogen-bonding and van der Waals inter­actions. Across the main absorption band, ɛ_1_(ω) undergoes anomalous dispersion, decreases sharply through zero and reaches a minimum near 16 eV before slowly recovering toward unity at higher energies.

**Figure 8:**
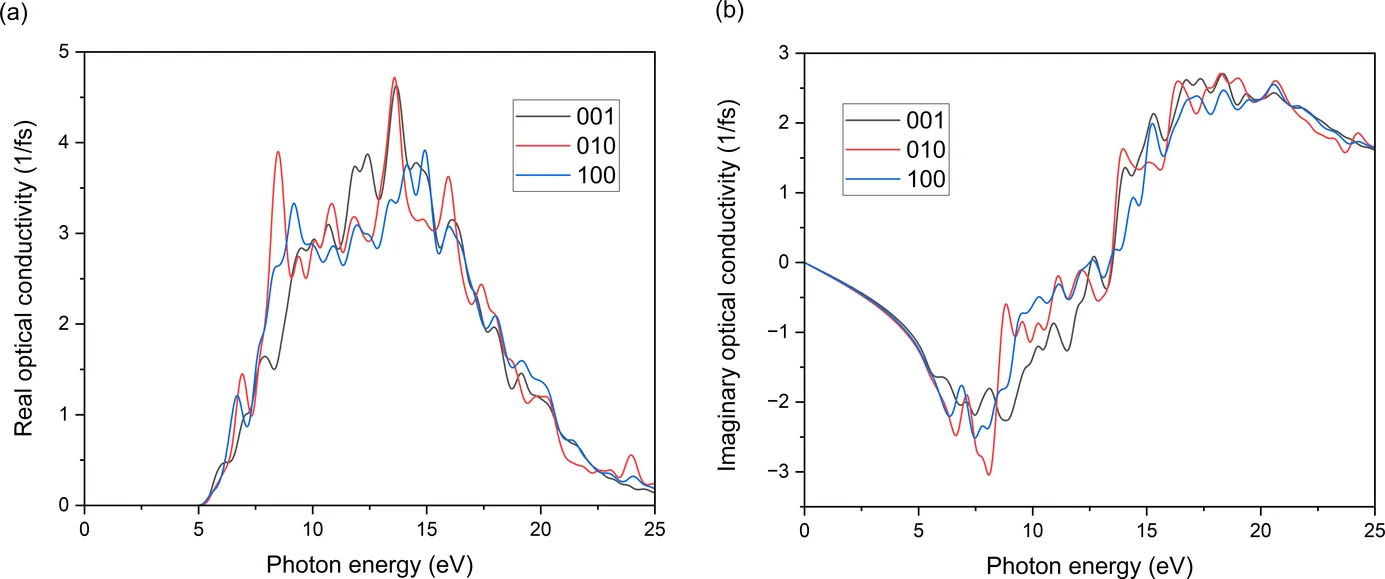
Optical conductivity for three polarizations.

Fig. 8 ▸ shows the real and imaginary components of the optical conductivity for three polarization directions. The real component becomes appreciable around 5 eV and exhibits its strongest peaks at approximately 13.6–14.9 eV, while the imaginary component displays a dispersive response with sign changes around 12–14 eV. Near 8.5 eV, the [010] response is approximately 2.40 times that for [001], indicating enhanced coupling to light polarized along the coordination chains. However, the dominant polarization varies with photon energy, showing that the chain-like structure does not produce a uniformly chain-dominated optical response. These results identify a polarization-sensitive ultraviolet region for future experimental investigation.

**Figure 9:**
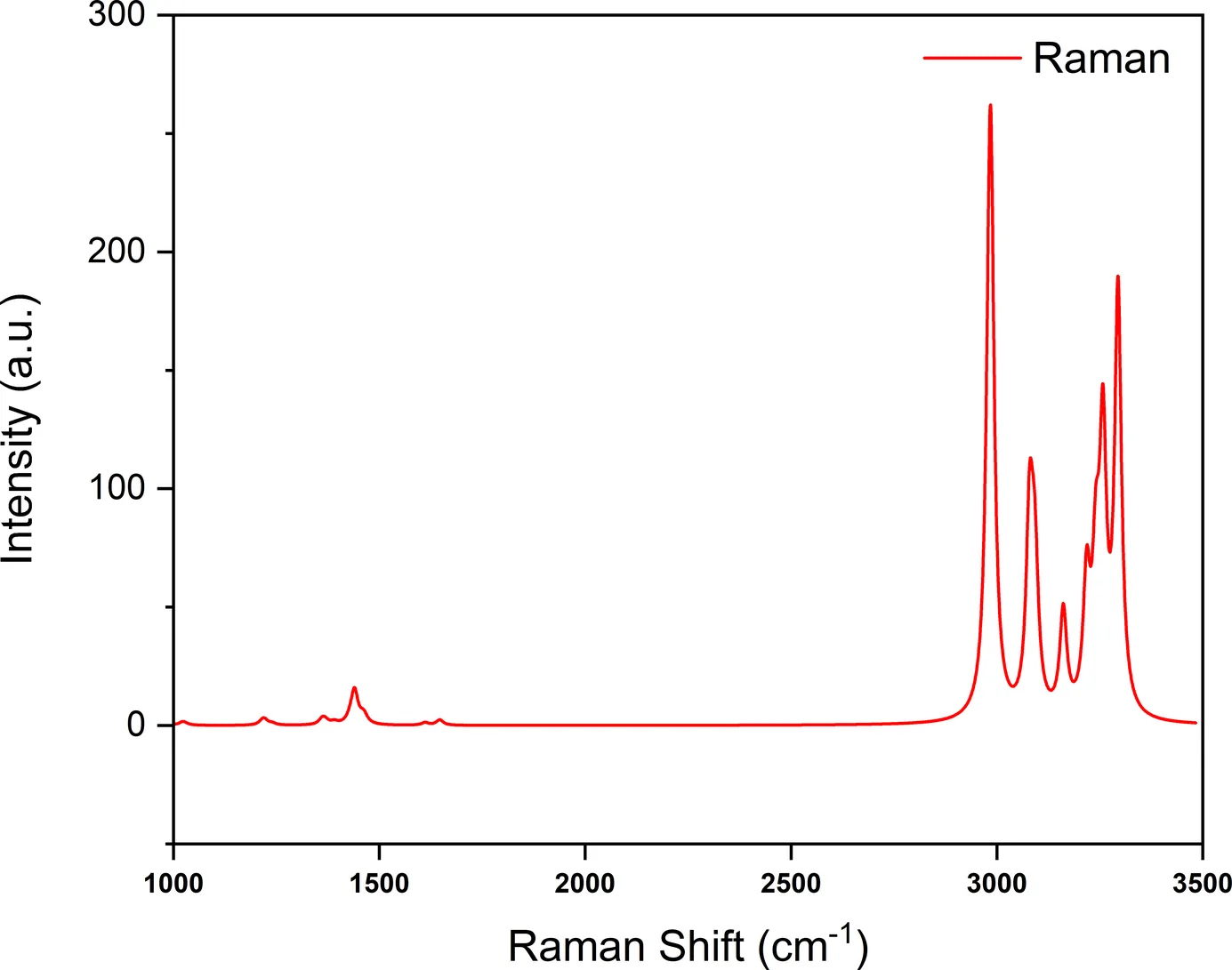
Simulated Raman spectrum.

The simulated Raman spectrum in the range 1000–3500 cm^−1^ is shown in Fig. 9 ▸. The most intense peaks at 2950-3050 cm^−1^ are assigned to the C—H stretching vibrations of the tri­methyl­ammonium methyl groups (Libowitzky, 1999 ▸). Of particular inter­est is the pair of peaks in the 3200–3400 cm^−1^ region, which is attributed to the O—H stretching of coordinated water and the N—H stretching of the tri­methyl­ammonium cation (Lin *et al.*, 2020 ▸). The positions of these peaks are consistent with the involvement of O—H and N—H groups in hydrogen bonding with chloride acceptors: the O—H stretches appear at frequencies moderately red-shifted relative to free water, in accord with the O—H⋯Cl hydrogen bonds (O⋯Cl = 3.1479 Å) identified crystallographically, and the N—H stretch likewise reflects the N—H⋯Cl inter­action (N⋯Cl = 3.3685 Å). These spectroscopic features provide independent computational support for the hydrogen-bonding network described in the supra­molecular analysis. Weak features around 1450–1500 cm^−1^ correspond to C—H bending modes (Snyder *et al.*, 1978 ▸).

## 6. Synthesis and crystallization

MnCl_2_·4H_2_O and (CH_3_)_3_NHCl were dissolved in deionized water in a 1:1 molar ratio. A small amount of hydro­chloric acid was added to maintain a weakly acidic environment. The mixture was stirred until homogeneous, and the resulting clear solution was left to stand in an open vessel at room temperature. Pale-pink block-shaped crystals suitable for single-crystal X-ray diffraction were obtained by slow evaporation over several days.

## 7. Refinement

Crystal data, data collection and structure refinement details are summarized in Table 2 ▸. H atoms bonded to carbon atoms were placed in calculated positions and refined as riding atoms, with C—H = 0.98 Å and *U*_iso_(H) = 1.5*U*_eq_(C) for the methyl groups. The H atom attached to N was also included in a calculated position and refined using a riding model, with *U*_iso_(H) = 1.2*U*_eq_(N). The H atoms of the coordinated water mol­ecule were included in the refinement using constrained positions, with *U*_iso_(H) = 1.5*U*_eq_(O).

## 8. Conflicts of inter­est

There is no conflict of inter­est here.

## Supplementary Material

Crystal structure: contains datablock(s) I. DOI: 10.1107/S2056989026010765/oo2019sup1.cif

Structure factors: contains datablock(s) I. DOI: 10.1107/S2056989026010765/oo2019Isup2.hkl

Supporting information file. DOI: 10.1107/S2056989026010765/oo2019Isup3.mol

CCDC reference: 2548320

Additional supporting information:  crystallographic information; 3D view; checkCIF report

## Figures and Tables

**Table 1:** Hydrogen-bond geometry (Å, °)

*D*—H⋯*A*	*D*—H	H⋯*A*	*D*⋯*A*	*D*—H⋯*A*
N1—H1⋯Cl2	1.00 (2)	2.67 (1)	3.3685 (19)	128 (1)
O1—H1*D*⋯Cl3	0.85 (1)	2.32 (1)	3.1479 (14)	165 (1)

**Table 2:** Experimental details

Crystal data	
Chemical formula	(C_3_H_10_)[MnCl_2_(HO_2_)_2_]Cl
*M*_r_	257.44
Crystal system, space group	Orthorhombic, *Pnma*
Temperature (K)	100
*a*, *b*, *c* (Å)	16.7931 (12), 7.4533 (5), 8.2357 (5)
*V* (Å^3^)	1030.81 (12)
*Z*	4
Radiation type	Mo *K*α
μ (mm^−1^)	2.01
Crystal size (mm)	0.36 × 0.32 × 0.14

Data collection	
Diffractometer	XtaLAB Mini II
Absorption correction	Multi-scan (*CrysAlis PRO*; Rigaku OD, 2018 ▸)
*T*_min_, *T*_max_	0.630, 1.000
No. of measured, independent and observed [*I* > 2σ(*I*)] reflections	6414, 1594, 1227
*R*_int_	0.030
(sin θ/λ)_max_ (Å^−1^)	0.709

Refinement	
*R*[*F*^2^ > 2σ(*F*^2^)], *wR*(*F*^2^), *S*	0.029, 0.072, 1.03
No. of reflections	1594
No. of parameters	57
H-atom treatment	H-atom parameters constrained
Δρ_max_, Δρ_min_ (e Å^−3^)	0.32, −0.38

Computer programs: *CrysAlis PRO* (Rigaku OD, 2018 ▸), *SHELXT* (Sheldrick, 2015*a* ▸), *SHELXL* (Sheldrick, 2015*b* ▸) and *OLEX2* (Dolomanov *et al.*, 2009 ▸).

## supplementary crystallographic information

### catena-Poly[trimethylammonium [[diaquamanganese(II)]-di-µ-chlorido] chloride] Crystal data

**Table:**

(C_3_H_10_N)[MnCl_2_(HO_2_)_2_]Cl	*D*_x_ = 1.659 Mg m^−^^3^
*M**_r_* = 257.44	Mo *K*α radiation, λ = 0.71073 Å
Orthorhombic, *P**n**m**a*	Cell parameters from 2016 reflections
*a* = 16.7931 (12) Å	θ = 2.4–30.0°
*b* = 7.4533 (5) Å	µ = 2.01 mm^−^^1^
*c* = 8.2357 (5) Å	*T* = 100 K
*V* = 1030.81 (12) Å^3^	Block, pink
*Z* = 4	0.36 × 0.32 × 0.14 mm
*F*(000) = 524	

### catena-Poly[trimethylammonium [[diaquamanganese(II)]-di-µ-chlorido] chloride] Data collection

**Table:**

XtaLAB Mini II diffractometer	*R*_int_ = 0.030
ω scans	θ_max_ = 30.3°, θ_min_ = 2.4°
Absorption correction: multi-scan (CrysAlis PRO; Rigaku OD, 2018)	*h* = −18→23
*T*_min_ = 0.630, *T*_max_ = 1.000	*k* = −10→10
6414 measured reflections	*l* = −11→11
1594 independent reflections	3 standard reflections every 150 reflections
1227 reflections with *I* > 2σ(*I*)	intensity decay: 0.4%

### catena-Poly[trimethylammonium [[diaquamanganese(II)]-di-µ-chlorido] chloride] Refinement

**Table:**

Refinement on *F*^2^	0 restraints
Least-squares matrix: full	Hydrogen site location: mixed
*R*[*F*^2^ > 2σ(*F*^2^)] = 0.029	H-atom parameters constrained
*wR*(*F*^2^) = 0.072	*w* = 1/[σ^2^(*F*_o_^2^) + (0.0349*P*)^2^] where *P* = (*F*_o_^2^ + 2*F*_c_^2^)/3
*S* = 1.03	(Δ/σ)_max_ = 0.001
1594 reflections	Δρ_max_ = 0.32 e Å^−^^3^
57 parameters	Δρ_min_ = −0.38 e Å^−^^3^

### catena-Poly[trimethylammonium [[diaquamanganese(II)]-di-µ-chlorido] chloride] Special details

**Table:**

Geometry. All esds (except the esd in the dihedral angle between two l.s. planes) are estimated using the full covariance matrix. The cell esds are taken into account individually in the estimation of esds in distances, angles and torsion angles; correlations between esds in cell parameters are only used when they are defined by crystal symmetry. An approximate (isotropic) treatment of cell esds is used for estimating esds involving l.s. planes.

### catena-Poly[trimethylammonium [[diaquamanganese(II)]-di-µ-chlorido] chloride] Fractional atomic coordinates and isotropic or equivalent isotropic displacement parameters (Å^2^)

**Table:**

	*x*	*y*	*z*	*U*_iso_*/*U*_eq_	Occ. (<1)
Mn1	0.500000	0.500000	0.500000	0.02283 (11)	
Cl1	0.60171 (3)	0.250000	0.50062 (7)	0.02673 (14)	
Cl2	0.39811 (3)	0.250000	0.56421 (8)	0.02891 (14)	
Cl3	0.41077 (4)	0.750000	−0.00188 (7)	0.03204 (16)	
O1	0.47692 (8)	0.46220 (16)	0.24016 (14)	0.0304 (3)	
H1D	0.514500	0.416176	0.184671	0.046*	
H1E	0.467192	0.561761	0.193600	0.046*	
N1	0.31714 (12)	0.250000	0.1900 (2)	0.0308 (5)	
H1	0.370071	0.250002	0.245686	0.037*	
C2	0.33240 (18)	0.250000	0.0103 (3)	0.0423 (7)	
H2A	0.363974	0.352680	−0.018105	0.064*	0.5
H2B	0.282514	0.254810	−0.046415	0.064*	0.5
H2C	0.360334	0.142510	−0.019375	0.064*	0.5
C1	0.27415 (12)	0.0848 (3)	0.2417 (2)	0.0431 (5)	
H1A	0.221696	0.081149	0.189685	0.065*	
H1B	0.267563	0.085600	0.359966	0.065*	
H1C	0.304908	−0.021171	0.209555	0.065*	

### catena-Poly[trimethylammonium [[diaquamanganese(II)]-di-µ-chlorido] chloride] Atomic displacement parameters (Å^2^)

**Table:**

	*U*^11^	*U*^22^	*U*^33^	*U*^12^	*U*^13^	*U*^23^
Mn1	0.0267 (2)	0.01811 (19)	0.02363 (18)	0.00153 (14)	−0.00167 (14)	−0.00124 (13)
Cl1	0.0216 (3)	0.0195 (3)	0.0391 (3)	0.000	0.0022 (2)	0.000
Cl2	0.0261 (3)	0.0238 (3)	0.0369 (3)	0.000	0.0035 (2)	0.000
Cl3	0.0399 (3)	0.0291 (3)	0.0272 (3)	0.000	0.0019 (2)	0.000
O1	0.0400 (7)	0.0259 (6)	0.0252 (6)	0.0060 (6)	0.0004 (5)	0.0000 (5)
N1	0.0249 (10)	0.0309 (11)	0.0366 (11)	0.000	−0.0078 (9)	0.000
C2	0.0428 (17)	0.0442 (17)	0.0401 (15)	0.000	−0.0005 (12)	0.000
C1	0.0383 (11)	0.0367 (11)	0.0542 (12)	−0.0047 (9)	−0.0053 (9)	0.0116 (9)

### catena-Poly[trimethylammonium [[diaquamanganese(II)]-di-µ-chlorido] chloride] Geometric parameters (Å, º)

**Table:**

Mn1—Cl1^i^	2.5276 (4)	N1—C2	1.502 (3)
Mn1—Cl1	2.5277 (4)	N1—C1^ii^	1.490 (2)
Mn1—Cl2	2.5845 (4)	N1—C1	1.490 (2)
Mn1—Cl2^i^	2.5845 (4)	C2—H2A	0.9600
Mn1—O1	2.1929 (11)	C2—H2B	0.9601
Mn1—O1^i^	2.1930 (11)	C2—H2C	0.9601
O1—H1D	0.8513	C1—H1A	0.9800
O1—H1E	0.8511	C1—H1B	0.9800
N1—H1	1.0000	C1—H1C	0.9800
			
Cl1^i^—Mn1—Cl1	180.0	H1D—O1—H1E	104.6
Cl1^i^—Mn1—Cl2^i^	85.151 (15)	C2—N1—H1	107.4
Cl1—Mn1—Cl2^i^	94.849 (15)	C1^ii^—N1—H1	107.4
Cl1—Mn1—Cl2	85.151 (15)	C1—N1—H1	107.4
Cl1^i^—Mn1—Cl2	94.849 (15)	C1^ii^—N1—C2	111.36 (13)
Cl2—Mn1—Cl2^i^	180.0	C1—N1—C2	111.36 (13)
O1^i^—Mn1—Cl1^i^	91.53 (3)	C1—N1—C1^ii^	111.5 (2)
O1—Mn1—Cl1	91.53 (3)	N1—C2—H2A	109.6
O1^i^—Mn1—Cl1	88.47 (3)	N1—C2—H2B	109.3
O1—Mn1—Cl1^i^	88.47 (3)	N1—C2—H2C	109.5
O1^i^—Mn1—Cl2	90.57 (4)	H2A—C2—H2B	109.5
O1—Mn1—Cl2	89.43 (4)	H2A—C2—H2C	109.5
O1—Mn1—Cl2^i^	90.57 (4)	H2B—C2—H2C	109.5
O1^i^—Mn1—Cl2^i^	89.43 (4)	N1—C1—H1A	109.5
O1—Mn1—O1^i^	180.0	N1—C1—H1B	109.5
Mn1^iii^—Cl1—Mn1	94.982 (19)	N1—C1—H1C	109.5
Mn1—Cl2—Mn1^iii^	92.269 (19)	H1A—C1—H1B	109.5
Mn1—O1—H1D	116.4	H1A—C1—H1C	109.5
Mn1—O1—H1E	111.2	H1B—C1—H1C	109.5

Symmetry codes: (i) −*x*+1, −*y*+1, −*z*+1; (ii) *x*, −*y*+1/2, *z*; (iii) −*x*+1, *y*−1/2, −*z*+1.

### catena-Poly[trimethylammonium [[diaquamanganese(II)]-di-µ-chlorido] chloride] Hydrogen-bond geometry (Å, º)

**Table:**

*D*—H···*A*	*D*—H	H···*A*	*D*···*A*	*D*—H···*A*
N1—H1···Cl2	1.00 (2)	2.67 (1)	3.3685 (19)	128 (1)
O1—H1*D*···Cl3	0.85 (1)	2.32 (1)	3.1479 (14)	165 (1)
